# Control of viral replication after cessation of HAART

**DOI:** 10.1186/1742-6405-8-6

**Published:** 2011-02-11

**Authors:** Ellen Van Gulck, Leo Heyndrickx, Lotte Bracke, Sandra Coppens, Eric Florence, Anne Buvé, Paul Lewi, Guido Vanham

**Affiliations:** 1 Virology unit, Department of Microbiology, Institute of Tropical Medicine, Antwerp (ITMA), Antwerp, Belgium; 2 HIV/STD unit, Department of Clinical Sciences, ITMA, Antwerp, Belgium; 3 STI/HIV Epidemiology and Control Unit, Department of Microbiology, ITMA, Antwerp, Belgium; 4 Department of Medicinal Chemistry, University of Antwerp (UA); Faculty of Medicine and Pharmacy, Free University of Brussels (VUB) and Department of Gynecology and Obstetrics, Catholic University of Leuven (KUL), Belgium; 5 Department of Biomedical Sciences, Faculty of Pharmaceutical, Veterinary and Biomedical Sciences, University of Antwerp and Faculty of Medicine and Pharmacy Free University of Brussels, Belgium

## Abstract

We describe two patients who did not experience a viral rebound after cessation of HAART which was initiated for progressive disease. CD4 T-cell count remained stable in one patient and progressively declined in the other, despite apparent viral control. We failed to identify any immune activation or genetic markers that could offer an explanation for this unusual "secondary controller" status. But their viruses are clearly less fit compared to viruses from rebounders.

## Introduction

Highly active antiretroviral treatment (HAART) has improved life expectancy and quality of life of HIV infected patients [1]. However, so far it is not possible to "cure" HIV infection mainly because latent reservoirs persist even in patients who are on effective combination treatment [2]. Cessation of HAART results in viral rebound within days or weeks and pre-treatment levels of viral load (VL) are typically reached within one year after treatment interruption [3,4].

In 2008 we identified a patient who, after stopping HAART, had a VL below the detection limit of 1,6log for more than 2 years. After searching the data base of 1,700 HIV infected patients under follow-up at the clinic of the Institute of Tropical Medicine, another patient was identified, who spontaneously controlled VL after cessation of HAART for at least 6 months. We labeled these two patients "secondary controllers" (SC) as opposed to "elite controllers" (EC) who spontaneously control viral replication but are treatment naive.

## Comparison between SC and "rebounders"

### Methods

In order to identify correlates of SC status, we compared clinical characteristics, viral factors, genetic traits and immune activation markers in the two SC and patients who rebound after treatment cessation. We used the following criteria to define SC and rebounders: (1) being HIV-1 infected for at least 3 years; (2) HAART started in the chronic progressive phase; (3) HAART discontinued for the first time; and (4) VL below the detection limit of 1.6log at treatment interruption. Pregnant women taking HAART to prevent mother-to-child transmission and patients who started treatment in the acute phase of infection were excluded. SC were defined as patients who met the above criteria and who kept their VL below 3log for at least 6 months after treatment cessation. In order to make sure that the SC were not taking antiretroviral drugs, plasma samples were tested for the presence of protease inhibitors (PI) and non nucleoside reverse transcriptase inhibitors at the Bioanalytical Laboratory of the Catholic University of Louvain.

The following parameters were assessed: HIV subtype, CD4 count, VL and number of days on HAART and were compared in SC and rebounders. In addition, we evaluated plasma markers of immune activation, including: neopterin; beta-2 microglobulin (B2M) (Demiditek, Germany); soluble CD14 (sCD14) (R&D, United Kingdom); and lipopolysaccharide (LPS) (Lonza, Belgium). These measurements were done on stored plasma samples taken before starting HAART (T1), during HAART (T2) and 6 months after stopping HAART (T3). Soluble markers in plasma were measured instead of activation markers on cells (Roberts AIDS 2010), because at our clinic only plasma samples of patients are stored.

In order to assess viral factors, the SC provided 100 ml of blood for purification of CD4+ T-cells. One part was used to extract DNA, as described elsewhere [5], for sequence analysis of Gag, Pol and Env. The other part was used to cultivate the virus [6].

In addition, we evaluated the relative replication capacity of P1. To assess viral fitness phytohemagglutinin (PHA) stimulated peripheral blood mononuclear cells (PBMC) from 3 healthy donors were infected at multiplicity of infection (MOI) 10^-3 ^with the viruses, IIIB and BAL as reference strains (NIH, Germantown, MD), and with virus isolated from SC patient 1 and from 4 different rebounders. P24 concentrations were measured by ELISA over time.

This study was approved by the Institutional Review Board of ITM and the Ethics Committee of the University Teaching Hospital in Antwerp. Informed consent was obtained from all patients.

### Results

When searching our database on 1700 patients in regular follow-up, we found that a total of 160 patients had stopped HAART for the first time, after a mean of 25 months of successful viral suppression. Treatment was stopped under medical guidance, mostly because of toxicity and/or because the patient wanted a drug holiday. At least two VL measurements after treatment cessation were available for 124 patients, which enabled us to classify them as "rebounders" or "SC". Median time to rebound (VL > 3log) was 7 weeks and median time to re-starting treatment was 4.5 months. Sixty-eight patients remained off treatment for at least 6 months. By that time all but 2 patients (the SC) had a VL that exceeded 3log. Both SC were negative on the drug assays.

## Case presentations

**Patient 1 **was diagnosed with HIV infection in 1985. In 1993 he started treatment with AZT, when his CD4 count was 228/μL. VL testing was unavailable at that time and no plasma samples from 1993 were available at the clinic in Antwerp. The first VL measurement dated from 1999 when the patient was already taking AZT monotherapy. It was 3.4 log and a PI based HAART regimen was started and continued till 2001, when the patient was switched to Nevirapine and 2 nucleoside reverse transcriptase inhibitors (NRTI's). He took this combination till treatment interruption in April 2005, when his CD4 count was 458/μl. In July 2005 the VL was 3.9log but from October 2005 until now it remained below the detection limit. The CD4 count, however, has been decreasing to 205/μL in February 2010 but the patient refuses to be restarted on treatment.

**Patient 2 **was found to be HIV positive in 1997, but was probably infected since 1991. In 1998 her CD4 count was 377/μL and her VL 4.6log. She was started on HAART with a PI based regimen. In 1999 she switched to Nevirapine and 2 NRTIs. Treatment was stopped in March 2006. The VL remained undetectable after treatment interruption, though in April 2009 there was a blip in the plasma VL of 2,9log, which was detected with the Cobas ampliprep/cobas Taqman (Roche, New Jersey, USA). The patient is still off treatment with a stable CD4 count above 1000/μL.

Table 1 summarizes the main clinical findings of the two SC and the 66 rebounders. All clinical parameters of SC, including VL before HAART and time on HAART, were within the range of the rebounders. Patient 2 carried HLA B3501, which is rather associated with rapid progression. Both SCs were homozygous for wild type CCR5. They also show similar levels of plasma immune activation during and after stopping HAART, as compared to the rebounders.

**Table 1:** Comparison between rebounders and secondary controllers

	Rebounders (n = 66)	Secondary controllers (n = 2)	
		**P1**	**P2**

Sex:			
Male	43	M	F
Female	23		

Age at start HAART (years)	37 (20-61)^a^	50 ^b^	45

**Clinical parameters of infection**			

Days since diagnosis	4563 (1529-8469)	7709	4191

Days on HAART	1364 (160-4063)	4257	2860

Days off HAART	541 (182-2468)	not restarted	not restarted

CD4 at start HAART	292 (23-804)	228	377

CD4 at stop HAART	698 (113-1669)	458	1326

VL before start HAART	4.98 (3.47-6.0)	3.4 (under AZT)	4.6 (treatment naïve)

VL 6 months after stopping HAART	4.69 (3.04-6.0)	<1.6	< 1.6

Months with undetectable VL under HAART	25 (1-98)	65	83

**HIV-1 subtype**			
	3 A	B	D
	17 B		
	1 D		
	2 H		
	3 CRF_01		
	4 CRF_02		
	1 CRF_36		
	1 CRF_D/F1		
	34 unknown		

**Markers of immune activation**			

sCD14 (ng/ml)			
T1	2066 (1682-2380)^c^	ND	1648
T2	2032 (1627-2499)	3037	3119
T3	2173 (1744-2740)	4269	1760

LPS (EU/ml)			
T1	4.04 (3.02-6.45)	ND	7.4
T2	4.17 (3.02-5.8)	3.41	3.36
T3	4.61 (3.41-5.35)	4.31	4.43

Neopterin (nmol/l)			
T1	8.15 (4.93-11.61)	ND	13.14
T2	18.22 (11.36-25.07)	9.35	17.76
T3	8.75 (5.37-17.1)	5.16	15.03

Beta-2 microglobulin (μg/ml)			
T1	448 (346-596)	ND	300
T2	252 (226-308)	336	212
T3	466 (344-566)	694	280

**HLA types**		B4101, B5201	B1402, B3501

ND: not done, because no plasma sample was available

T1 = before start of HAART; T2 = during HAART; T3 = 6 months after treatment cessation:

^a ^Rebounders: median (min-max)

^b ^For secondary controllers number is given

^c ^Rebounders: median (IQR)

Virus measured as p24 in culture supernatant of purified CD4+ T-cells from patients under HAART, typically emerges after 3 to 6 days. In contrast, in patient 1 it emerged on day 21, while in patient 2 virus culture failed despite three attempts. In 2009, however, the latter patient had a blip in plasma virus suggesting that the virus is not completely defective. Moreover the sequences of Gag, Pol and Env in the two SC did not show any obvious defects, and no CD8 T-cell associated escape mutations could be detected. Remarkably, phylogenetic analysis of env from patient 1 revealed the presence of two related but distinct viruses. Both viruses were lacking the lentivirus lytic peptides (LLP) 1 region, but could still be cultured.

**Figure 1:**
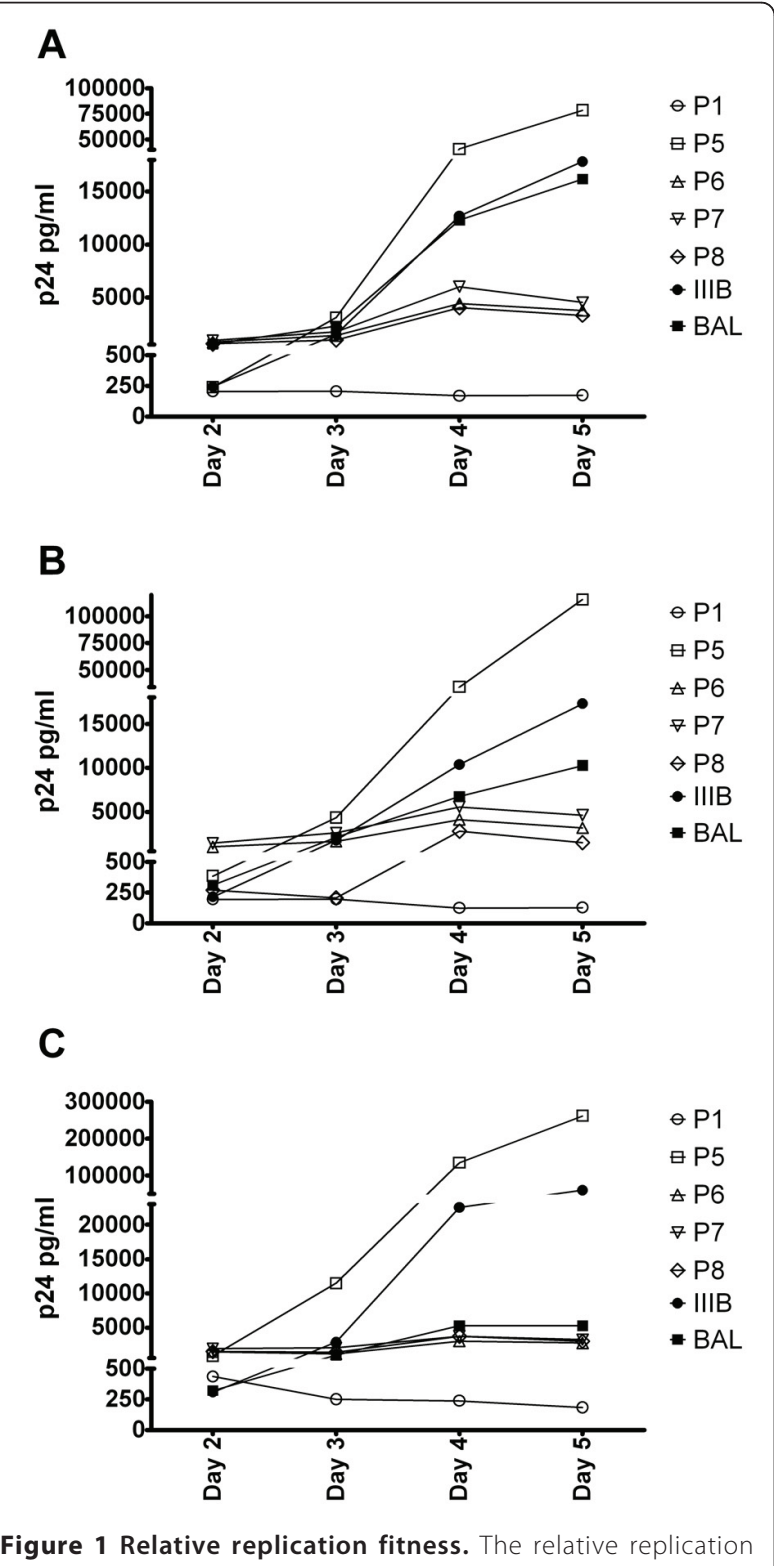
**Relative replication fitness. **The relative replication fitness was determined in PHA blasts from 3 healthy donors by measuring the p24 production produced by HIV reference strains BAL and IIIB or patient viruses (P1 is secondary controller, P5-P8 is from rebounders). (A): p24 production in ng/ml in donor 1. (B): p24 production in ng/ml in donor 2. (C): p24 production in ng/ml in donor 3.

Autologous viruses from patient 1 could be cultured making it possible to investigate whether the virus of this patient was less fit in comparison to reference strains or virus of 4 different rebounders. Figure 1 clearly shows that the virus of the SC replicates less as compared to the reference strains and viruses of rebounders since the amount of p24 produced in PHA stimulated PBMC remains much lower over time in comparison to viruses of rebounders and the reference strain.

## Discussion

We identified two HIV-1 infected patients with an exceptional clinical course after stopping HAART. One patient kept stable and high CD4 T-cell counts for more than 4 years after treatment cessation, while the other patient had progressive disease with decreasing CD4 T-cell counts. Such discrepancy of evolution has also been described in elite controllers [7-9].

To our knowledge there is only one previous study that had identified patients who maintained an undetectable viral load for several months after treatment cessation. Skiest et al mentioned 2 patients who had an undetectable viral load for 6 months after treatment was stopped as part of a study on the effects of treatment interruption. However, they just mention them without comparing them with patients whose viral load rebounded [10]. We are aware of only one study that has explored factors predictive of "non-rebounder" status [11]. Bedimo et al defined non-rebounders as patients who had a VL below 3,7log at least 6 months after treatment interruption while we defined SC as patients who had a VL below 3log. In Bedimo's cohort 27% of patients were non-rebounders and peak VL prior to HAART was predictive of non-rebounder status. In contrast, the SC we identified had a VL prior to starting HAART that was in the range of VL of rebounders which we defined as having a VL > 3 log 6 months after HAART cessation. In our study VL before HAART initiation seemed thus not to be associated with control of viral replication after treatment cessation.

One possible explanation for the SC status is that deleterious mutations have been induced in the virus by HAART. However, sequencing of the major structural genes of the 2 available viruses did not reveal any obvious defects. Virus could be cultivated in patient 1 only, but patient 2 had a spontaneous "in vivo" blip. We hypothesize that these replication competent viruses are less fit and/or are kept under control by the patient's immune system. Indeed, as shown in Figure 1, virus from patient 1 was less fit than the viruses from rebounders and the two reference strains BAL and IIIB. The fact that the virus from patient 2 could not be cultivated in vitro under conditions that easily allow virus growth in all other patients is also an implicit proof of lower fitness.

Markers of immune activation are more disturbed in patients with rapid progression and closer to normal in EC [12,13]. Neopterin and β2 microglobulin have also been shown to predict disease progression, especially in late stage HIV-1 infection [14-16]. However, we did not find any differences between SC and rebounders, in levels of immune activation before initiating therapy, during therapy and after treatment cessation, suggesting that immune activation does not play a role in SC status.

Research on EC is very active because these patients may give clues for novel approaches to the development of a preventive HIV vaccine. Research on immune function in the secondary controllers in this study is ongoing; as we believe that they might provide new insights about correlates of protection and be useful for the development of therapeutic vaccines.

## Consent

Written informed consent was obtained from the patients for publication of this case report. A copy of the written consent is available for review by the Editor-in -chief of this journal.

## Competing interests

The authors declare that they have no competing interests.

## Authors' contributions

**EVG **performed all culture experiments and ELISAs for immune activation, contributed to the study design, data analysis and interpretation of the data; and wrote the paper. **LH **was responsible for the molecular biological aspects of this study and performed all sequence analyses. **LB**: performed the experiments to investigate the replicative fitness.

**SC **performed all DNA isolations and amplifications. **EF **was responsible for the clinical aspects of the study. He identified the patients, abstracted the clinical information and recruited the patients in the study. **AB **contributed to the study concept, and the writing of the project proposal and the paper. **PL **performed all the statistical data analyses. **GV **supervisor and principal investigator of the project, contributed to the study design, data analysis and interpretation of the data. All authors have read and approved the final manuscript.

## Authors' information

**EVG **is a postdoc at the Institute of Tropical medicine. During her PhD she was trying to develop a therapeutic vaccination against HIV based on dendritic cells electroporated with mRNA encoding HIV Gag. In this studies patients on HAART were vaccinated with these DC. After vaccination it is the goal that they stop treatment and control the virus. Therefore these patients are so special because they can do it without vaccination. If we can find out why than we know to which parameter we have to look for protection in a therapeutic vaccination. **AB, EF, GV **are clinicians. EF is really working in the clinic and seeing patients. AB and GV are more interested in research. For EF is it also important if we can unravel why these patients control the virus after treatment interruption, maybe more patients can go on drug holiday.
